# A rapid MALDI-TOF mass spectrometry workflow for *Drosophila melanogaster* differential neuropeptidomics

**DOI:** 10.1186/1756-6606-6-60

**Published:** 2013-12-27

**Authors:** Joseph P Salisbury, Kristin J Boggio, Yun-Wei A Hsu, Jeniffer Quijada, Anna Sivachenko, Gabriele Gloeckner, Paul J Kowalski, Michael L Easterling, Michael Rosbash, Jeffrey N Agar

**Affiliations:** 1 Department of Biology, Brandeis University, Waltham, MA 02453, USA; 2 Department of Chemistry and Volen Center for Complex Systems, Brandeis University, Waltham, MA 02453, USA; 3 Howard Hughes Medical Institute, National Center for Behavioral Genomics, and Department of Biology, Brandeis University, Waltham, MA 02451, USA; 4 Depts of Chemistry and Chemical Biology and Pharmaceutical Sciences and Barnett Institute of Chemical and Biological Analysis, Northeastern University, 140 The Fenway, Boston, MA 02115, USA; 5 Institute of Zoology, Department of Developmental Biology, University of Regensburg, Regensburg 93040, Germany; 6 Bruker Daltonics Inc., Billerica, MA 01821, USA; 7 Current Address: Department of Neurology, University of Massachusetts Medical School, Worcester, MA 01655, USA; 8 Current Address: Center for Integrative Brain Research, Seattle Children’s Research Institute, Seattle, WA 98105, USA

**Keywords:** Neuropeptidomics, MALDI-TOF, *Drosophila melanogaster*, Neuropeptides, Pigment dispersing factor, Tandem mass spectrometry, NPLP1, Neuropeptide-like precursor 1

## Abstract

**Background:**

Neuropeptides are a diverse category of signaling molecules in the nervous system regulating a variety of processes including food intake, social behavior, circadian rhythms, learning, and memory. Both the identification and functional characterization of specific neuropeptides are ongoing fields of research. Matrix-assisted laser desorption/ionization-time of flight mass spectrometry (MALDI-TOF MS) analysis of nervous tissues from a variety of organisms allows direct detection and identification of neuropeptides. Here, we demonstrate an analysis workflow that allows for the detection of differences in specific neuropeptides amongst a variety of neuropeptides being simultaneously measured. For sample preparation, we describe a straight-forward and rapid (minutes) method where individual adult *Drosophila melanogaster* brains are analyzed. Using a MATLAB-based data analysis workflow, also compatible with MALDI-TOF mass spectra obtained from other sample preparations and instrumentation, we demonstrate how changes in neuropeptides levels can be detected with this method.

**Results:**

Over fifty isotopically resolved ion signals in the peptide mass range are reproducibly observed across experiments. MALDI-TOF MS profile spectra were used to statistically identify distinct relative differences in organ-wide endogenous levels of detected neuropeptides between biological conditions. In particular, three distinct levels of a particular neuropeptide, *pigment dispersing factor*, were detected by comparing groups of preprocessed spectra obtained from individual brains across three different *D. melanogaster* strains, each of which express different amounts of this neuropeptide. Using the same sample preparation, MALDI-TOF/TOF tandem mass spectrometry confirmed that at least 14 ion signals observed across experiments are indeed neuropeptides. Among the identified neuropeptides were three products of the neuropeptide-like precursor 1 gene previously not identified in the literature.

**Conclusions:**

Using MALDI-TOF MS and preprocessing/statistical analysis, changes in relative levels of a particular neuropeptide in *D. melanogaster* tissue can be statistically detected amongst a variety of neuropeptides. While the data analysis methods should be compatible with other sample preparations, the presented sample preparation method was sufficient to identify previously unconfirmed *D. melanogaster* neuropeptides.

## Background

Neuropeptides are a large and diverse class of signaling molecules that affect numerous processes, including behavior, development, heart rate, metabolism, and reproduction [1,2]. These peptides, mostly exerting their role by acting upon G-protein coupled receptors [3], can function as classical hormones, localized neurohormones [4], at muscles and glands, and synaptically, where they can modify the postsynaptic response to classical, fast-acting neurotransmitters [5]. Studies of neuropeptide function that cross a wide variety of aspects of behavior and development have been particularly productive in the model organism *Drosophila melanogaster*[3,6-14], which benefits from exceptional genetic manipulation tools developed for the study of the molecular mechanisms of development and behavior. In model insects such as *D. melanogaster*, *Apis mellifera* (honeybee), and *Tribolium castaneum* (red flour beetle), 30–40 genes have been consistently identified as encoding neuropeptides [8,15,16], with each gene product potentially producing multiple different mature neuropeptides. To become active, neuropeptides often require multiple post-translational modifications, such as proteolysis and amidation, which are difficult to infer from a genome and necessitate that putative neuropeptides be directly identified in organisms, often using mass spectrometry-based methods. Bioinformatics studies have predicted as many as 156 neuropeptides encoded by 33–119 putative neuropeptide genes in *D. melanogaster*, and a total of 76 neuropeptides from 21 genes have been detected experimentally [3,17]. The sensitivity of MS-based methods has allowed for detection and identification of neuropeptides from specific nervous system regions and cellular populations across the developmental lifespan of *D. melanogaster*[1,6,7,9,18,19] permitting precise temporal and spatial localization to be ascribed to various neuropeptides.

While great strides have been made towards comprehensive identification of *D. melanogaster* neuropeptides, functional characterization is lacking for many. For example, the majority of the peptides derived from the *D. melanogaster* gene neuropeptide-like precursor 1 (NPLP1) remain “orphaned” without an identified receptor and/or physiological function [20]. Quantitative neuropeptidomics provides a discovery tool for ascertaining functional significance of neuropeptides, with goals of monitoring and quantifying changes in levels of multiple neuropeptides in response to experimental perturbations such as those eliciting complex behavioral responses. For example, isotope labeling followed by UPLC-ESI-QTOF has been used to quantify ~50 of known *Apis mellifera* brain peptides in the context of foraging, revealing molecular connections between the regulation of food intake in individual insects and this social behavior, as well as distinctions between nectar and pollen gathering [21]. Isotopic labeling from extracts using MALDI-TOF MS combined with direct tissue MALDI imaging has been used to provide complementary information regarding changes in the expression of an array of neuropeptides during feeding in both the brain and pericardial organ of the crab *Cancer borealis*[22]. A label-free LC-Orbitrap approach was employed to analyze extracts from hypothalamus and striatum from rats, using higher-energy collision dissociation and electron transfer dissociation fragmentation to identify more than 1700 endogenous peptides, revealing upregulation of orexigenic and anorexigeneic neuropeptides in animals fed on a high-fat/high-sucrose diet [23]. Direct access to quantitative neuropeptidomics techniques, however, is often limited to laboratories equipped with a considerable array of specialized instrumentation, reagents, and personnel, preventing these methods from being more routinely utilized by those studying *D. melanogaster* development and behavior. Thus, we sought to develop a rapid method for performing differential expression neuropeptidomics studies utilizing *D. melanogaster* that does not require specialized reagents or advanced MS instrumentation. Furthermore, we wanted to present a data analysis workflow utilizing software that could preprocess and statistically analyze MS data regardless of instrument manufacturer.

Here we present a *D. melanogaster* sample preparation method that, when analyzed with matrix-assisted laser desorption/ionization time-of-flight mass spectrometry (MALDI-TOF MS), reliably detects an abundance of ions in the peptide mass range, 14 of which we subsequently confirmed by MALDI-TOF/TOF tandem mass spectrometry (MS/MS) to be *D. melanogaster* neuropeptides. Amongst the neuropeptides we identified by MS/MS fragmentation were three peptides derived from the NPLP1 gene not identified previously in the literature. Utilizing a MATLAB-based spectra preprocessing workflow, we demonstrate the ability to statistically detect differences in the expression of a specific neuropeptide, amongst all the ions we simultaneously observe, without isotopic labeling using MALDI-TOF MS.

## Results and discussion

### Straight-forward on-target peptide extraction provided adequate signal quality for MALDI-TOF MS profiling as well as targeted MALDI-TOF/TOF MS/MS

We set out to develop a sample preparation strategy for comparing neuropeptidomes from *D. melanogaster* that: could be performed in minutes, thus preserving labile biomolecules; could detect a large number of ions simultaneously, ideally with abundant enough signal to confidently identify using MALDI-TOF/TOF MS/MS; did not require extensive utilization of specialized reagents or equipment beyond a standard benchtop MALDI-TOF MS (at least for detection); and that utilized, ideally, only a single fly brain as an individual sample for statistical comparisons. The overall sample preparation we used consisted of dissection of individual *D. melanogaster* brains followed by their direct placement onto a steel MALDI target, an on-target wash, and matrix application. As a dissection medium, a modified dissection saline consisting of 7.5 g/L NaCl, 0.2 g/L KCl, 0.2 g/L CaCl_2_, and 0.1 g/L NaHCO_3_ in MilliQ water (pH 7.2) [6] was chosen as it yields a high number of detected ion signals and a relatively low baseline compared with dissection solutions with higher concentrations of salts. Solutions of fructose [24], Tris, and ammonium bicarbonate were evaluated as wash steps at various concentrations, with 100 mM ammonium bicarbonate producing the most abundant number of ions detected in the peptide mass range. Included in the detectable peaks with this sample preparation was *m/z* 1972.0, which we believed and later confirmed was the neuropeptide *pigment-dispersing factor* (PDF). This relatively low abundance peptide was used in subsequent experiments (see below) to demonstrate that known differences in relative levels of neuropeptides could be detected with this technique. With the ammonium bicarbonate wash, however, excess ammonium bicarbonate on the MALDI target after drying from the wash and subsequent matrix deposition was occasionally observed, which can interfere with homogenous crystallization of MALDI matrix and decrease the quality of acquired spectra (~10% of spectra). Alternatively, we found washing by dipping the brain in ammonium bicarbonate after dissection, but prior to placement on the MALDI target, as an alternative to the on-target wash. Data shown in the profiling experiments comparing flies with varying of PDF were acquired from samples prepared with the on-target wash, with spectra only acquired from samples that were not contaminated by excess ammonium bicarbonate.

Finally, various concentrations of 2,5-dihydroxybenzoic acid (DHB) and α-cyano-4-hydroxycinnamic acid (CHCA) were tested, ranging from 10–50 mg/mL and 5–10 mg/mL, respectively, as a matrix for MALDI-MS analysis, with 10 mg/mL CHCA providing the most reliable and highest quality spectra in terms of number of peaks with signal-to-noise (S/N) ratios greater than 6. During MS acquisition we noted that the entire crystalline matrix surface across a given spotted sample on the target did not yield homogeneous spectra. Specifically, both the surface of the brain itself and the edges of the MALDI spot yielded poor S/N ratios, leaving a “halo” region of high S/N spectra around the tissue (Additional file 1: Figure S1), consistent with what has long been observed in MALDI analyses of biological peptides from tissue samples [25]. We attempted to improve homogeneity by trituration of the matrix solution; by homogenizing the brain on-target using a pipette tip or pressing with a cover slip; and by using a microcentrifuge tube homogenizer, but all of these procedures resulted in reduced S/N spectra. Overall, minimal mechanical perturbation of the brain was found to be important for achieving optimal quality spectra from single brains. Spectra of homogenized samples could be improved using a reversed phase “ZipTip,” but this required ~20 brains and added an additional step. As a result of the lack of MALDI spot homogeneity, we cannot be sure that peptides from all regions of the brain are extracted with identical efficiency. While this might prevent analysis of specific neuropeptides using this method, our primary goals of being able to detect simultaneously a variety of neuropeptides from various regions of the brain (as well as determine distinct differences in the levels of these neuropeptides when making experimental comparisons, with particular focus on PDF, as described below) were achieved.

**Figure 1:**
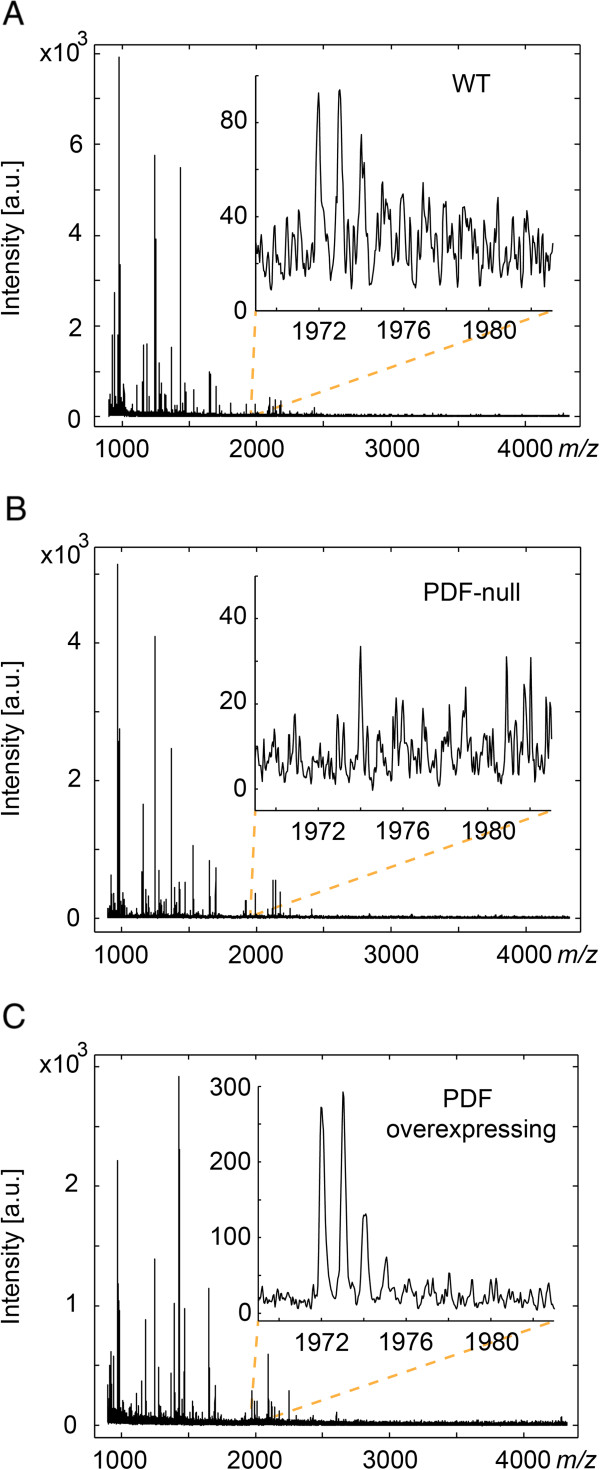
**Representative raw spectra from WT flies and flies lacking expression of, and overexpressing, PDF. A)** Spectra acquired from WT flies included many well resolved peaks with ion signal at *m/z* 1972.0 (inset, [M + H]^+^ of PDF) generally resolvable, although often with low S/N. **B)** Spectra acquired from PDF-null flies were similarly rich in features, but without discernible ion signal at *m/z* 1972.0 (inset). **C)** Spectra acquired from PDF overexpressing flies, while of overall similar quality to those obtained from WT flies, had the ion signal at *m/z* resolved with far greater S/N.

Overall, raw spectra acquired with the final method yielded spectra with 37 ± 9.6 (mean ± S.D.) isotopically resolved peak distributions of S/N greater than 6 within the *m/z* 900–4000 range prior to any preprocessing (Figure 1A). Of importance was the ion signal at *m/z* 1972.0, confirmed in experiments below to correspond to the monoisotopic [M + H]^+^ of the neuropeptide PDF. Using the *yellow white* (*yw*, henceforth referred to as WT or wild-type flies) fly strain as a control strain, the PDF signal from *m/z* 1972.0 was often detectable in spectra from individual fly brains, but often only barely distinguishable from noise (Figure 1A inset). To demonstrate this sample preparation technique could be used in a differential neuropeptide profiling experiment to statistically identify differences in neuropeptide-derived ion signals, including neuropeptide-derived ion signals with low S/N ratios like PDF, flies expressing varying levels of PDF were obtained and analyzed. In particular, the *pdf*^*01*^ fly strain [14] (henceforth referred to as “PDF-null” flies) was used as a mutant strain lacking any expression of mature PDF and flies overexpressing PDF throughout the adult nervous system (referred subsequently in the text as “PDF overexpressing” flies) were generated using the GAL4-UAS binary expression system [26] by crossing the pan-neuronal elav-GAL4 driver line with a UAS-*Drm-pdf* line (see Methods section). Indeed, in spectra from individual flies, the ion signal corresponding to PDF was never observed in samples from PDF-null flies (Figure 1B), and was almost always observed with S/N > 6 in spectra from PDF overexpressing flies (Figure 1C). From this, we determined an experimental design that would permit changes in PDF to be statistically identified to validate this sample preparation method as a means for differential profiling of neuropeptides.

### Preprocessing of spectra permitted statistical identification of distinct detection levels of ions corresponding to neuropeptide PDF

The most accurate methods of MS-based quantification generally involve the use of isotopologue standards [21]. Relative quantitation of neuropeptide standards can be achieved over a thousand-fold concentration range on a MALDI-TOF mass spectrometer using isotopic labeling after careful selection of appropriate data acquisition parameters [27]. Our method could be adapted to isotope dilution using any of the strategies demonstrated to quantify neuropeptides in single neurons or neuron clusters [28]. However, in the current study, we show it is also possible to detect several distinct levels of expression of a neuropeptide in a label-free profiling approach using preprocessed MALDI-TOF MS spectra acquired from individual *D. melanogaster* brains. To achieve this, MALDI-TOF MS spectra from brains of WT flies were compared to PDF-null flies and PDF overexpressing flies. All brains were dissected from flies within a two hour window centered at Zeitgeber time 2 after entrainment to a 12:12-hour light–dark cycle at 25°C. Within an experiment, fly brains were analyzed individually, with multiple fly brains per genotype analyzed to permit statistical analysis of differences observed. Two full experiments were performed in order to further analyze the experimental reproducibility. In one experiment where the relative expression levels of PDF were compared, spectra were acquired from 10 WT*,* 7 PDF-null, and 9 PDF overexpressing *D. melanogaster* brains. In a second experiment, spectra were acquired from 9 WT, 7 PDF-null, and 9 PDF overexpressing *D. melanogaster* brains. Differences in sample numbers between experiments occurred due to spectra not being acquired from certain prepared samples judged to have poor crystallization, potentially from excess ammonium bicarbonate.

**Figure 2:**
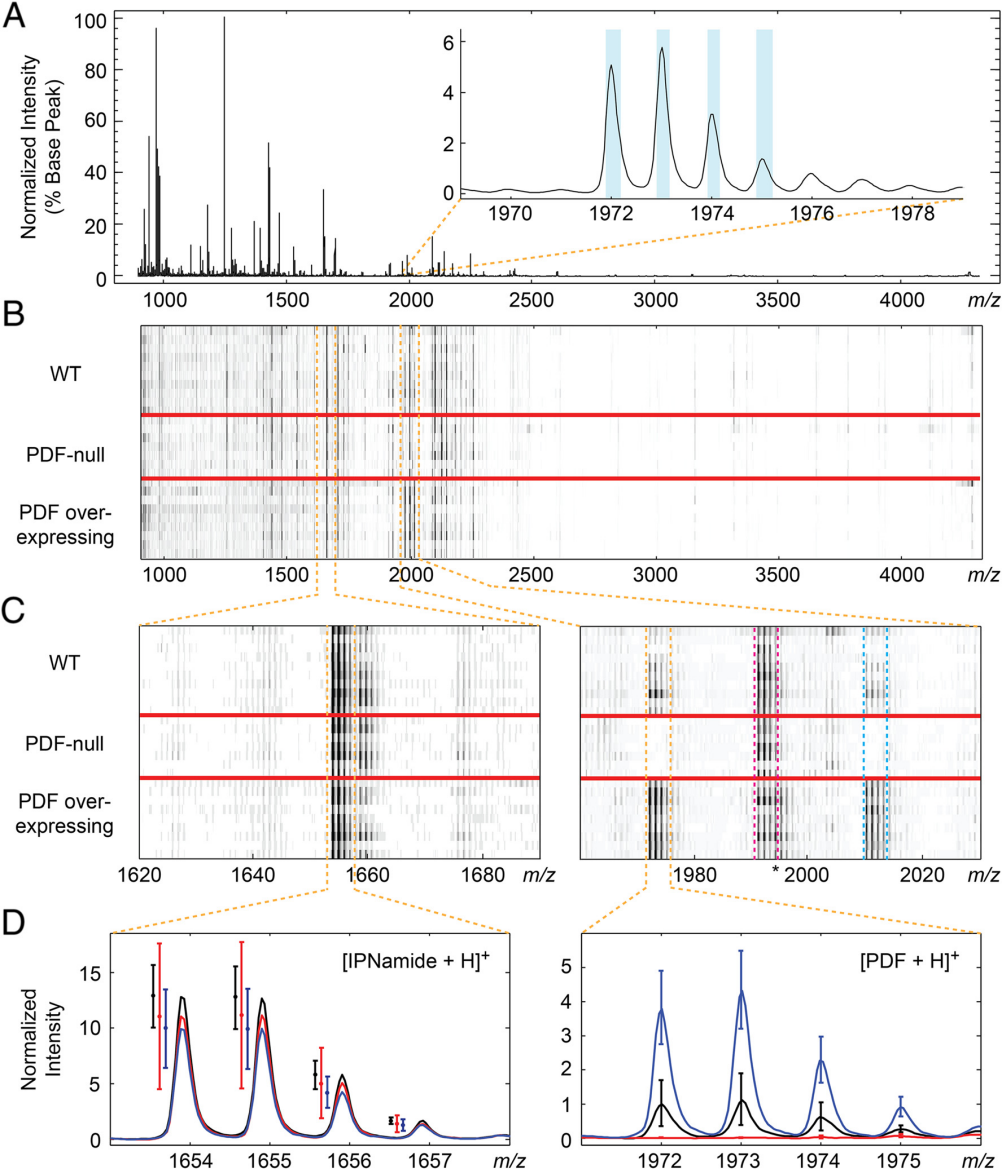
**Overview of spectra acquired within an experiment where changes in ion signals corresponding to PDF were detectable statistically. A)** Total average spectrum used to define peaks detected within an experiment. The isotopic distribution with monoisotopic peak at *m/z* 1972.0 is shown in inset. Peak bins detected using our described criteria are highlighted in blue. **B)** Pseudogel view after preprocessing of all spectra acquired in a particular replicate experiment. **C)** Zoomed in pseudogel view at *m/z* regions containing the isotopically resolved [M + H]^+^ of IPNamide (monoisotopic *m/z* 1653.9, left panel) and the [M + H]^+^ of PDF (monoisotopic *m/z* 1972.0, boxed off in orange dashed lines, right panel) and the [M + K]^+^ of PDF (monoisotopic *m/z* 2010.0, boxed off in blue dashed lines, right panel). Also seen in right panel is the isotopic distribution with monoisotopic peak at *m/z* 1991.0 (boxed off in the dashed magenta lines), corresponding to the truncated and amidated variant of NPLP13 (QRAamide). Peaks trailing from this distribution observed in the PDF overexpressing flies likely corresponds to the [M + Na]^+^ of PDF (monoisotopic *m/z* 1994.0, marked by an asterisk) which was not resolved enough in the total average spectrum to be detected as a distinct isotopic distribution. **D)** Averages of preprocessed spectra within experimental conditions at two *m/z* regions (red = PDF-null, black = WT, blue = PDF overexpressing). The [M + H]^+^ of IPNamide (left, 95% confidence intervals shown to the left of each peak for clarity), which was found not to vary significantly across conditions (Kruskal-Wallis ANOVA raw p-value = 0.22), is compared with the [M + H]^+^ of PDF (right), which was detected to be significantly different across the three conditions (Bonferroni-adjusted p-value = 0.0017).

In order to use mass spectra obtained from individual fly brains for the purposes of differential neuropeptide expression analysis, a spectra preprocessing workflow [29] was employed that includes spectrum denoising, baseline correction, and normalization (see Methods section for full description of preprocessing). Peaks bins were then chosen from peaks identified in a total average spectrum, which was calculated from all spectra (after preprocessing) acquired across the three conditions (Figure 2A). Deisotoping criteria was applied in order to identify isotopically resolved peak distributions from the peaks detected in the total average spectrum. The inset of Figure 2A highlights the peak bins that were considered to be a single isotopomer distribution with a monoisotopic peak at *m/z* 1972.0 (*i.e.* PDF). While over 300 peaks were identified in the total average spectra of the two replicate experiments described here, after applying deisotoping criteria, exactly 57 isotopically resolved distributions were identified in either experiment, with 52 ion masses observed common to both replicate experiments (Additional file 2: Table S1).

Peak bins corresponding to isotopically resolved ion signals were used to query across all preprocessed spectra (Figure 2B). From this, a value for every ion signal isotopically resolved in the total average spectrum was assigned in the individual sample spectra regardless of whether that signal was detectable in any particular sample spectrum. Identifying ion signals in a total average spectrum, as opposed to individual spectra, may reduce the sensitivity of feature detection. For example, ion signals only detectable in a particular set of samples might be “averaged out” in the total average spectrum. However, by using a common set of peak bins across all spectra, some value can be established for every ion signal for the purpose of statistical comparisons, avoiding a “missing value” problem for peaks not otherwise “detectable” in a given spectrum. Summed deisotoped intensities were then compared with the non-parametric Kruskal-Wallis analysis of variance (ANOVA) test (α = 0.01). We adjusted for multiple comparisons using the straight-forward and conservative Bonferroni correction.

In the experiment comparing spectra from individual brains of 10 WT*,* 7 PDF-null, and 9 PDF overexpressing *D. melanogaster*, there was sufficient power to detect three significant differences between groups, including the distributions with monoisotopic peaks at *m/z* 1972.0 (Bonferroni-adjusted *p*-value = 0.0017, confirmed by MALDI-TOF/TOF MS/MS to be the [M + H]^+^ of PDF), *m/*z 2010.0 (Bonferroni-adjusted *p*-value = 0.0014, inferred to be the [M + K]^+^ of PDF), and *m/z* 1203.6 (Bonferroni-adjusted *p*-value = 0.0099, *m/z* currently unassigned). Post-hoc analysis of the PDF species confirmed the difference was significant between all three groups, with levels increasing in the logical order (Tukey’s least significant difference procedure, α = 0.05). Figure 2C and D illustrate differences in ion signals corresponding to PDF compared to ion signal that did not vary significantly, *m/z* 1653.9 (Raw *p*-value = 0.22, subsequently identified as the [M + H]^+^ of IPNamide). Post-hoc analysis of *m/z* 1203.6 suggested detection of this isotope distribution was significantly reduced in the PDF overexpressing condition. In the second full profiling experiment performed, spectra were acquired from 9 WT, 7 PDF-null, and 9 PDF overexpressing flies, with only two isotope distributions being significantly different, the [M + H]^+^ of PDF (Bonferroni-adjusted *p*-value = 0.0097) and the [M + K]^+^ of PDF (Bonferroni-adjusted *p*-value = 0.0043). The significance of the difference in the unidentified *m/z* 1203.6 was not replicated in this experiment (Bonferroni-adjusted *p*-value = 0.1142, see Additional file 2: Table S1 for full results of statistics from both experiments). Post-hoc analysis of the PDF isotope distributions in this experiment again confirmed that PDF was detected at distinct levels across the three conditions in the expected order.

**Figure 3:**
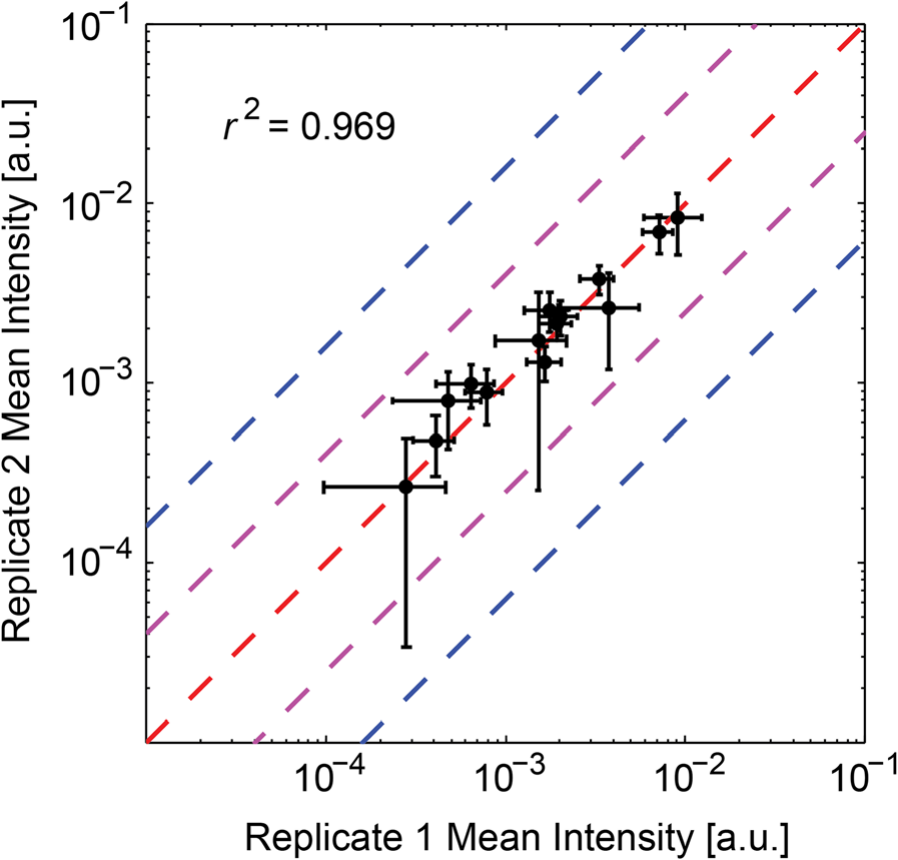
**Comparison of mean preprocessed ion intensities and 95% confidence intervals between replicate experiments of WT flies for the fourteen isotope distributions identified by MALDI-TOF/TOF MS/MS to be neuropeptides.** Linear regression between the mean values of the fourteen isotope distributions gave an *R*^2^ = 0.969. The dashed red line shows where mean intensity values should be had they been identical between the two repeat experiments. The dashed purple and blue lines show where the log_2_ ratio between two conditions would be 1 or 2, respectively. 95% confidence intervals in either experiment generally were within 1 log_2_ ratio.

To further evaluate the general reproducibility of this method, the correlation between intensities of isotopically resolved ion signals detected in both experiments was examined. Focusing specifically on the fourteen ion signals later confirmed by MS/MS to be neuropeptides (see next subsection), the correlation between replicates of the mean intensities of these signals within the WT condition was high, with *R*^2^ = 0.969 (Pearson correlation, Figure 3). In the other two experimental conditions, the correlation between experiments was still generally high, with *R*^2^ = 0.894 for the PDF-null condition and *R*^2^ = 0.871 for the PDF overexpressing condition (Additional file 2: Table S2). Expanding this analysis out to all 52 isotopically resolved signals observed in both experiments, the correlation was generally high in each condition, with *R*^2^ = 0.957 for the WT condition, *R*^2^ = 0.848 for the PDF-null condition, and *R*^2^ = 0.914 for the PDF overexpressing condition (Additional file 2: Table S3).

Neuropeptide PDF was potentially detected as both an [M + H]^+^ at *m/z* 1972.0 and an [M + K]^+^ at *m/z* 2010.0 (as well as a [M + Na]^+^ at *m/z* 1994.0, which was not full resolved in the total average spectrum but is clearly present in the PDF overexpressing condition, marked by an asterisk in Figure 2). While both the [M + H]^+^ and [M + K]^+^ were found to be reproducibly significantly different between the three genotypes examined, the ratio of the relative intensities of these two forms of PDF ions were not necessarily consistent across an experiment. In particular, we evaluated the correlation between the intensities of the [M + H]^+^ and [M + K]^+^ of neuropeptide PDF within experiments (Additional file 2: Table S4). In the second experiment, there was a high correlation between the [M + H]^+^ and [M + K]^+^ of neuropeptide PDF in both the WT and PDF overexpressing conditions (*R*^2^ = 0.957 and 0.951, respectively). There was no correlation between the [M + H]^+^ and [M + K]^+^ signals in the PDF-null samples from the second experiment (*R*^2^ = 0.022) as would be expected given there should be no actual PDF signal in those samples. In the first experiment, however, while there remained a strong correlation between the [M + H]^+^ and [M + K]^+^ signals in the WT samples (*R*^2^ = 0.802), there was very low correlation between the [M + H]^+^ and [M + K]^+^ within the PDF overexpressing samples (*R*^2^ = 0.110), suggesting that perhaps residual potassium was unevenly distributed amongst samples in this experiment. Despite this inconsistency in the relative abundance of the potassium adduct of PDF compared with the [M + H]^+^, the differences in PDF levels between genotypes examined in this experiment were substantial enough to be detected whether either ionized form of PDF was considered. However, as this is a potentially confounding source of variability, it is important to consider whether detected changes in ion signal may be due shifts to different adduct ions, potentially from biases introduced during sample preparation such as inconsistent washing with ammonium bicarbonate.

### MS/MS analyses confirm many ions detected are neuropeptide including three novel identifications

**Figure 4:**
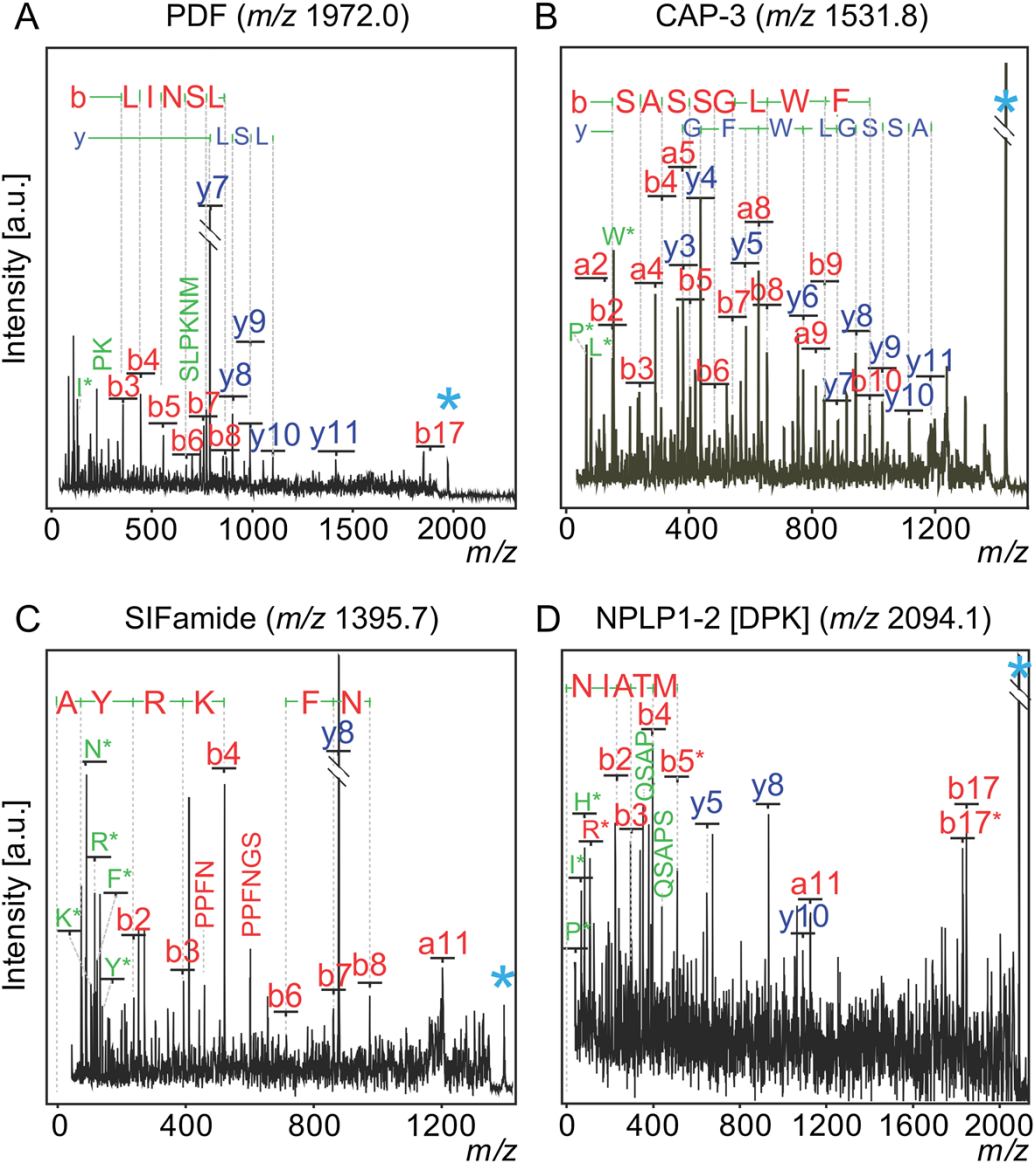
**MS/MS-based identification of PDF, CAP-3, SIFamide, and a novel NPLP1-derived peptide, NPLP1-2 (DPK peptide), from single fly brains.** MS/MS major peak assignments and corresponding sequence tags for: **A**, PDF; **B**, CAP-3; **C**, SIFamide; **D**, NPLP1-2 (DPK). Light blue asterisk denotes the precursor ion. y-type ions are shown in dark blue. b- and a-type ions shown in red. Iminium ions are shown in green and denoted by the one letter symbol of their respective amino acid followed by an asterisk. Internal fragments (b-y) are shown in green and denoted by their amino acid sequence. Similar results were obtained in duplicate and triplicate analyses.

After utilizing MALDI-TOF MS profiling to identify differences in particular ions, the next logical step would be to identify what those ions are, preferably without additional sample preparation. Indeed, MALDI-TOF/TOF MS/MS data obtained using this sample preparation permitted identification of multiple neuropeptides, including PDF (Figure 4, Table 1). This was reassuring given that one concern might have been that the ions we observed were not in fact neuropeptides, but rather, for example, proteolytic fragments from abundant proteins. The rapidity of this sample preparation, preserving labile biomolecules, may be one reason we do not have this problem. In-source/post-source decay could also yield ions that obscure neuropeptide detection. Thus far, however, none of the 14 molecular ions analyzed by MS/MS appear to result from the breakdown of larger molecules during MS analysis.

**Table 1:** Peaks observed in MALDI-TOF MS profile spectra confirmed to be neuropeptides by MALDI-TOF/TOF MS/MS fragmentation

**Obs. *m/z *** **(Calc. *m/z*)**	**Precursor (UniProtKB Entry name)**	**Peptide name**	**Peptide sequence**	**Previous ref.**
925.481* (925.436)	FMRF_DROME	PDNFMRFamide	R.PDNFMRFa.G	[1,2,7,9,30]
974.592 (974.589)	SNPF_DROME	RLRF peptide 2	R.SPSLRLRFa.G	[1,2,9,18,19,30]
1182.578 (1182.573)	FMRF_DROME	DPKQDFMRFamide (FMRFamide 2)	R.DPKQDFMRFa.G	[1,2,7,9,19,30]
1247.658 (1247.653)	NEMS_DROME	Dromyosuppressin (TDVDHVFLRFamide)	R.TDVDHVFLRFa.G	[1,2,7,9,18,19,30]
1395.748 (1395.753)	Q59E62_DROME	SIFamide (IFa-1)	A.AYRKPPFNGSIFa.G	[1,2,9,18,19]
1430.745 (1430.754)	CP2B_DROME	CAP-3^AA2-AA15^	T.GPSASSGLWFGPRLa.G	[1,2,9,19,30]
1471.791 (1471.773)	NPLP1_DROME	MTYamide peptide [NPLP1 (MTY)]	R.YIGSLARAGGLMTYa.G	[1,2,7,9,18,19]
1531.812 (1531.802)	CP2B_DROME	CAP-3 (CAPA-3)	R.TGPSASSGLWFGPRLa.G	[1,2,9,19,30]
1534.819 (1534.834)	NPLP1_DROME	NPLP1-4 [NPLP1 (VQQ)]	R.NLGALKSSPVHGVQQ.K	[1,2,18,19]
1653.907 (1653.908)	NPLP1_DROME	IPNamide [NPLP1 (IPN)]	R.NVGTLARDFQLPIPNa.G	[1,2,7,9,18,19,30]
1972.015 (1972.017)	PDF_DROME	Neuropeptide PDF	R.NSELINSLLSLPKNMNDAa.G	[19]
1991.044 (1991.043)	NPLP1_DROME	NPLP1-3^AA1-AA18^ [QRAamide]	R.NVAAVARYNSQHGHIQRAa.G	Novel
2094.091 (2094.088)	NPLP1_DROME	NPLP1-2 [NPLP1 (DPK)]	R.NIATMARLQSAPSTHRDPK.R	Novel
2249.130 (2249.128)	NPLP1_DROME	NPLP1-3 [NPLP1 (GAE)]	R.NVAAVARYNSQHGHIQRAGAE.K	Novel

Listed are *D. melanogaster* neuropeptides identified in this study. The final three listings are believed to be novel. Reported observed *m/z*’s are the average of the *m/z*’s observed between the two replicate experiments MALDI-TOF MS experiments described. All observed *m/z*’s listed were determined to be monoisotopic [M + H]^+^’s of listed neuropeptides based on the calculated monoisotopic [M + H]^+^ (shown in parentheses below observed *m/z*). Truncated peptides are denoted by superscripts showing amino acids present from the annotated peptide sequence (i.e. NPLP1-3^AA1-AA18^ is missing the final three residues of the annotated NPLP1-3 sequence, also shown). Peptide sequences include pre- and post- cleavage residues separated from sequences by a period. C-terminal amidation is denoted by an “a” at end of the peptide sequence. Abbreviations are listed in Table 2 legend. *m/z 925.481 likely corresponds to the convolution of PDNFMRFamide (monoisotopic [M + H]^+^ = 925.435) and Drostatin-3 (Ast-A3, monoisotopic [M + H]^+^ = 925.489, see Table 2), which were detected as separate peaks with MALDI-FTICR-MS (Figure 6), hence the comparatively larger *m/z* error.

**Figure 5:**
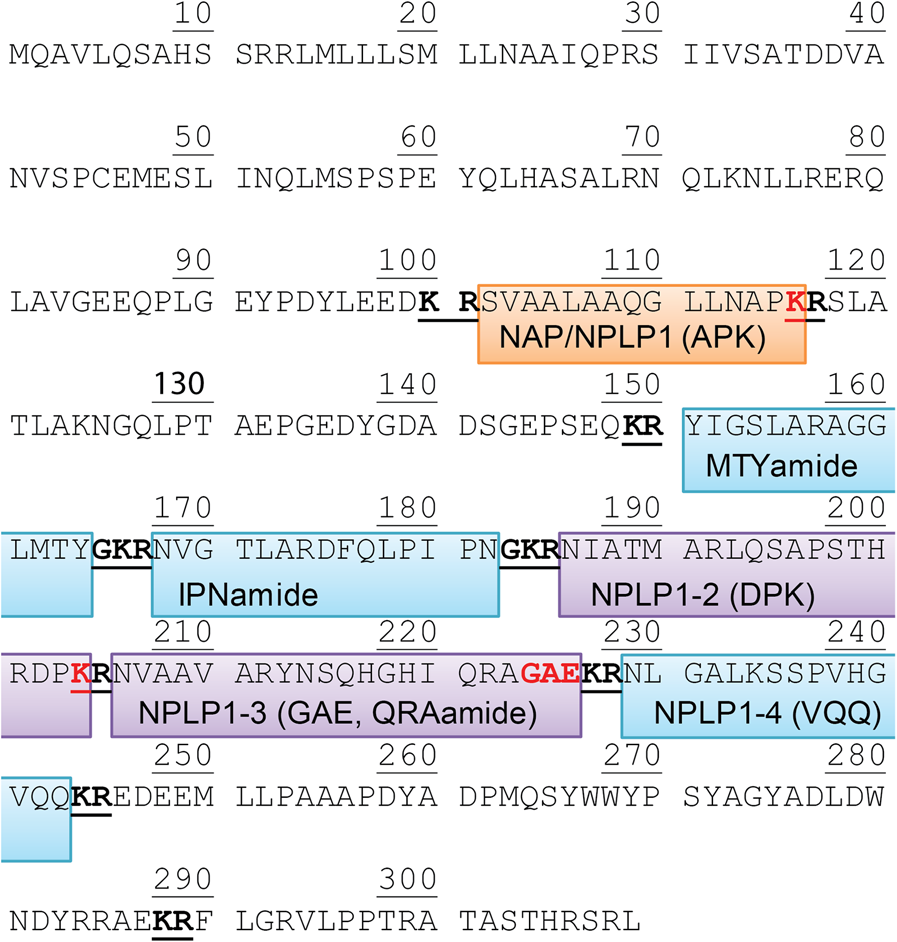
**NPLP1 sequence with peptides identified in this study and/or previously.** In blue are three peptides (MTYamide, IPNamide and VQQ), which were identified both previously and in this study with MALDI-TOF/TOF MS/MS. In purple are the regions containing three novel molecules we detected and identified. One peptide we identified corresponded to the predicted NPLP1-2, which we detected with an intact C-terminal lysine (DPK peptide), marked “K” in red. Also identified were two distinct peptides corresponding to the predicted NPLP1-3, with (GAE) and without (QRAamide) a C-terminal GAE sequence marked in red. The NPLP1-3 variant without the GAE fragment was observed to be amidated (QRAamide). We also detected and tentatively assigned, but not identified by fragmentation, an ion mass corresponding to the peptide outlined in orange, which has been reported with (as APK peptide) and without (as NAP peptide) a C-terminal K (marked in red). Our tentative assignment corresponded to the peptide with C-terminal K intact. Dibasic residue cleavage sites are shown underlined and bolded. Additional peptides that have been predicted but not identified are not explicitly highlighted.

Among identifications obtained were three previously predicted but unconfirmed peptides originating from the neuropeptide-like precursor 1 gene (NPLP1, Figure 5). Included in these identifications were two variants of the predicted NPLP1-3, without and without a C-terminal GAE (with the peptide lacking GAE being C-terminally amidated), and a peptide corresponding to the predicted NPLP1-2 but with a C-terminal lysine intact (NIATMARLQSAPSTHRDPK, or following previous convention, DPK peptide for short). It is possible the unamidated, glycine-extended NPLP1-3 (NVAAVARYNSQHGHIQRAGAE) is a precursor to the truncated and amidated variant (NVAAVARYNSQHGHIQRAa, or QRAamide), which may be the functional form of this peptide [31,32]. Four other peptides derived from NPLP1 have been detected previously [1,7,33]. We also identify three of these, IPNamide, MTYamide, and VQQ, using MS/MS and tentatively assigned the fourth, APK peptide (measured monoisotopic *m/z* 1423.814/theoretical 1423.827). While the VQQ peptide of NPLP1 (NPLP1-4) has been identified as a ligand for receptor guanylate cyclase Gyc76C, serving a role in modulating the innate immune IMD pathway in response to salt stress [20], precise functions for the remaining NPLP1-derived peptides are unknown. The ability to monitor and detect changes in these peptides in response to various *D. melanogaster* experimental paradigms will hopefully provide insight into their potential significance.

**Figure 6:**
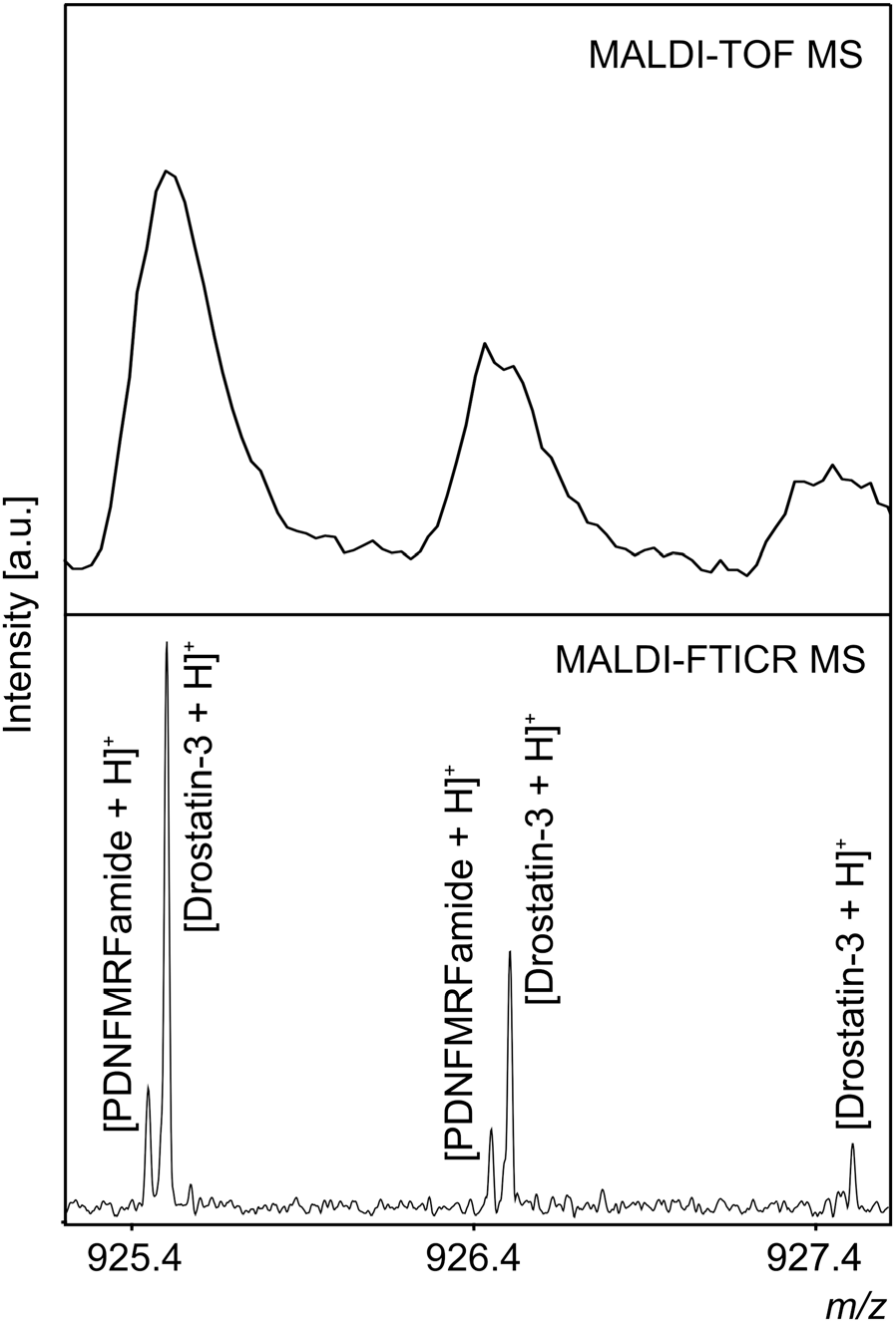
**High resolution MALDI-FTICR MS resolves neuropeptides PDNFMRFamide and Drostatin-3 (Ast-A3), convoluted in MALDI-TOF mass spectra.** Through the use of MALDI-FTICR MS, we were able to resolve *m/z* 925.481 from the MALDI-TOF MS (top) as being the convolution of two neuropeptides. PDNFMRFamide, identified by MALDI-TOF/TOF MS/MS fragmentation, was observed in MALDI-FTICR MS (bottom) as 925.43564 (theoretical monoisotopic [M + H]^+^ = 925.43594). A second peak at *m/z* 925.49004 was tentatively assigned to correspond to Drostatin-3 (Ast-A3, calculated [M + H]^+^ = 925.48994).

Curiously, *m/z* 925.481, determined by MALDI-TOF/TOF MS/MS to be PDNFMRFamide (monoisotopic [M + H]^+^ = 925.435) was of a slightly higher mass error (50 ppm) compared with other identified peaks (average mass error = 4 ppm). Subsequent analysis with MALDI-Fourier transform ion cyclotron resonance (FTICR) MS, which has substantially higher resolving power compared with MALDI-TOF MS, confirmed this peak was actually a convolution of PDNFMRFamide and a peak tentatively assigned as Drostatin-3 (Ast-A3, monoisotopic [M + H]^+^ = 925.489, Figure 6), hence the comparatively larger m/z error. Including Drostatin-3, 30 additional molecular ions observed in at least some MALDI-TOF profiling spectra have been tentatively assigned by mass matching to be neuropeptides (Table 2). Of these 30 molecular ions, 19 were abundant enough to be detected (using our stringent deisotoping criteria) in both experiments presented here. This implies that perhaps at least 63% of the molecular ions detected in our MALDI-TOF MS profiling experiments are neuropeptides [(14 neuropeptides detected in both experiments identified with MS/MS + 19 additional peaks detected in both experiments tentatively assigned to neuropeptides)/52 isotopically resolved ion signals detected in both experiments], suggesting this method is highly specific for this type of biomolecule.

**Table 2:** Peaks observed in MALDI-TOF MS profile spectra tentatively assigned to known neuropeptides by mass matching

**Obs. *m/z *** **(Calc. *m/z*)**	**Precursor (UniProtKB Entry name)**	**Peptide name**	**Peptide sequence**	**Previous ref.**
915.399 (915.415)	FMRF_DROME	SDNFMRPamide (FMRFamide 9)	R.SDNFMRFa.G	[1,2,7,9,30]
925.481 (925.490)	ALLS_DROME	Drostatin-3 (Ast-A3)	R.SRPYSFGLa.G	[1,2,7,9,18]
**936.463** (936.473)	TACHY_DROME	TK-3 (dTK-3, APTGFTGMRamide)	R.APTGFTGMRa.G	[1,2,18]
**942.584** (942.589)	TACHY_DROME	TK-2 (dTK-2, APLAFVGLRamide)	K.APLAFVGLRa.G	[1,2,18]
953.529 (953.521)	ALLS_DROME	Drostatin-1 (Ast-A1)	R.VERYAFGLa.G	[9,18]
961.486 (961.504)	TACHY_DROME	TK-5 (dTK-5, APNGFLGMRamide)	R.APNGFLGMRa.G	[1,2,18,19]
**982.622** (982.606)	SNPF_DROME	sNPF-3 (KPQRLRWamide)	R.KPQRLRWa.G	[18]
985.592 (985.588)	SNPF_DROME	sNPF-4 (KPMRLRWamide)	R.KPMRLRWa.G	[18,19]
996.556* (996.572*)	SNPF_DROME	RLRF peptide 2	R.SPSLRLRFa.G	[1,2,9,18,19,30]
1005.557 (1005.512)	FMRF_DROME	SAPQDFVRSamide (FMRFamide 12)	R.SAPQDFVRSa.G	[9,19,30]
**1015.603** (1015.605)	CP2B_DROME	CAP-2 (CAPA-2)	K.ASGLVAFPRVa.G	[1,2,7,9,30]
1065.560 (1065.552)	TACHY_DROME	TK-1 (dTK-1, APTSSFIGMRamide)	R.APTSSFIGMRa.G	[1,2,9,18]
1076.560 (1076.568)	TACHY_DROME	TK-4 (dTK-4, APVNSFVGMRamide)	R.APVNSFVGMRa.G	[1,2,9,18,19]
**1112.529** (1112.520)	FMRF_DROME	TPAEDFMRFamide (FMRFamide 7)	R.TPAEDFMRFa.G	[1,2,7,9,19,30]
1157.554 (1157.549)	CORZ_DROME	Corazonin^AA3-AA11^	T.FQYSRGWTNa.G	[1,2,30]
**1161.551** (1161.558)	AKH_DROME	AKH Peptide (+ C-term GK)	C.pQLTFSPDWGK.R	[9,19,30]
**1186.538** (1186.511)	DSK_DROME	Drosulfakinin-1 (DSK-1)	R.FDDYGHMRFa.G	[9,19]
1253.616 (1253.617)	MIP_DROME	Drostatin-B5 (Ast-B4)	R.DQWQKLHGGWa.G	[1,2,7,18,19]
**1276.696** (1276.680)	ALLS_DROME	Drostatin-4 (Ast-A4)	R.TTRPQPFNFGLa.G	[1,2,7,9,18,19]
**1294.681** (1294.673)	CP2B_DROME	CAP-1 (CAPA-1)	R.GANMGLYAFPRVa.G	[1,2,7,9,30]
1324.727 (1324.717)	Q59E62_DROME	IFa-2	A.YRKPPFNGSIFa.G	[19]
**1329.786** (1329.787)	SNPF_DROME	RLRF peptide 1	K.AQRSPSLRLRFa.G	[9,18,30]
**1369.644** (1369.629)	CORZ_DROME	Corazonin (Crz Peptide)	G.pQTFQYSRGWTNa.G	[1,2,7,9,19,30]
**1423.814** (1423.827)	NPLP1_DROME	NAP peptide (+ C-term K) [NPLP1 (APK)]	R.SVAALAAQGLLNAPK.R	[7,9,18,19]
1452.744* (1452.736*)	CP2B_DROME	CAP-3^AA2-AA15^	T.GPSASSGLWFGPRLa.G	[1,2,9,19,30]
**1603.841** (1603.835)	MIP_DROME	Drostatin-B3 (Ast-B3)	R.RQAQGWNKFRGAWa.G	[9,18,19]
**1658.677** (1658.665)	DSK_DROME	Drosulfakinin-2 (DSK-2)	R.GGDDQFDDYGHMRFa.G	[9]
**1741.940** (1741.962)	LCK_DROME	Leucokinin (DLK)	R.NSVVLGKKQRFHSWGa.G	[1,2,7,9,19,30]
2009.982* (2009.973*)	PDF_DROME	Neuropeptide PDF	R.NSELINSLLSLPKNMNDAa.G	[19]
**2176.201** (2176.188)	CP2B_DROME	CAP Propeptide 3	R.GDAELRKWAHLLALQQVLD.K	[30]

Ion masses observed to be isotopically resolved in both replicate experiments are underlined and bolded. As in Table 1, these reported observed *m/z*’s are the average of the *m/z*’s observed between the two replicate MALDI-TOF MS profiling experiments described. Truncated peptides are denoted by superscripts showing amino acids present from the annotated peptide sequence as described in Table 1. Peptide sequences include pre- and post- cleavage residues separated from sequences by a period. *All observed *m/z*’s listed are predicted to be monoisotopic [M + H]^+^’s of listed neuropeptides based on the calculated monoisotopic [M + H]^+^ (shown in parentheses below observed *m/z*) with the exceptions of *m/z* of *m/z* 996.572 and 1452.736 are [M + Na]^+^’s and *m/z* 2009.982 is an [M + K]^+^. C-terminal amidation is denoted by “a”, N-terminal pyroglutamation is denoted by “p”. Abbreviations: Ast – allatostatin, AKH – adipokinetic hormone, CAP - Cardioacceleratory peptide, NPLP1 - Neuropeptide-like precursor 1, PDF - Pigment-dispersing factor, sNPF-Small neuropeptide F, TK - Tachykinin-related peptide.

## Conclusion

We acknowledge the rigor of isotopic labeling-based approaches to quantitative mass spectrometry and are aware of confounds inherent to MALDI MS-based quantification (*i.e.* differences in ionization efficiency of analytes, ion suppression effects, etc.). These notwithstanding, there are numerous examples of label-free MALDI-TOF MS-based methods providing informative semi-quantitative results [34-36]. Indeed, MALDI-TOF MS neuropeptide peak detection alone has been sufficient to distinguish particular cell types and tissues in *Drosophila*[6,19]. Given the benefits of MALDI-TOF MS in terms of relative instrument expense and maintenance, as well as the ease of sample preparation and data acquisition, MALDI-TOF MS, even in instances when isotopic labeling is prohibitive, can serve as a valuable discovery tool, particularly when discovery of relatively more pronounced differences is an acceptable achievement (as opposed to absolute quantification). Here we present a rapid, label-free MALDI-TOF MS-based method and data analysis workflow that permits detection of differences in specific neuropeptides amongst a panel being monitored, using individual *D. melanogaster* brains as sample points. The described MATLAB-based preprocessing workflow and statistical analysis is compatible with other MALDI-TOF MS sample preparation techniques, including those previously described by other groups that have obtained spectra of excellent quality using other *D. melanogaster* tissues, including more specific *D. melanogaster* brain regions such as the antennal lobe [18] and individual cells [6]. The relatively straight-forward sample preparation method described here was sufficient to enable both detection of distinct levels of neuropeptide expression, as well as identify previously unconfirmed neuropeptides. Similar to other discovery-based methodologies, we strongly recommend differences in ion intensities detected with the described technique be validated by an independent method (such as more quantitative MS and/or immuno-based methods). However, we believe this validation effort is worth the additional time given the relative ease of the initial discovery procedure.

## Methods

### Fly stocks

*D. melanogaster* were reared on standard medium and raised under 12:12-hour light–dark conditions at 25°C. Flies were dissected between one to three hours after lights-on (two hour window centered at Zeitgeber time 2) when PDF expression levels are high [37]. The pan-neuronal elav-GAL4 driver line was Bloomington stock #8760 (Bloomington Drosophila Stock Center at Indiana University, Bloomington, IN, USA).

### Generation of UAS-Drm-pdf transgenic flies

Full-length *D. melanogaster pdf-*cDNA (*Drm-pdf-*cDNA) was kindly provided by Jeffrey C. Hall [38] and initially cloned into the pBluescript II SK (+/-) vector. To generate the UAS-*Drm-pdf* construct, the *Drm-pdf-*cDNA was then subcloned into the appropriate sites of the polylinker of the pUAST vector [26]. The pUAST vector contains a P-element for which the transposase gene has been replaced by the sequences of the GAL4-specific UAS, the hsp70 TATA-box, the mini-*white* gene and the SV40 polyadenylation signal. The construct has been confirmed by direct sequencing using vector specific primers. Transgenic flies were generated by germline transformation following standard protocols. Briefly, the pUAST-*Drm-pdf*-cDNA construct and the transposase gene-containing helper plastmid *pUChs*ΠΔ2-3 [39] were co-injected into *Drosophila w*^1118^ embryos using standard injection protocols. Two homozygous transgenic fly lines with different chromosomal localization of the construct (line no. 77: III chromosome) were obtained.

### D. melanogaster *brain dissection and on-target extraction*

Flies were dissected in a modified insect dissection saline (NaCl 7.5 g/L, KCl 0.2 g/L, CaCl_2_ 0.2 g/L, NaHCO_3_ 0.1 g/L; pH 7.2) [6] and a single dissected fly brain was transferred with non-locking forceps (Dumont Tweezers #5, 11 cm, 0.025 × 0.005 mm tip, World Precision Instruments) to a stainless steel MALDI target. Excess dissecting saline was removed during the transfer of the brain. While the brain was still on the forceps, the forceps were touched about 2 mm from the fly brain in the void space between the two arms of the forceps, using a KimWipe™ paper (Kimberly-Clark Worldwide, Inc.), which wicked away excess liquid. An on-target wash of 1.0 μL of 100 mM ammonium bicarbonate was performed using a pipettor to add and aspirate the solution. 0.5 μL of 10 mg/mL CHCA in 50% (v/v) acetonitrile, 0.1% (v/v) formic acid was then directly pipetted onto the brain and allowed to dry before MALDI-MS analysis.

### MALDI-TOF MS analysis of single dissected fly brains

Mass spectra were acquired on a microflex MALDI-TOF mass spectrometer (Bruker Daltonics Inc., Billerica, MA) equipped with a 337 nm N_2_ laser. Positive ion mass spectra were acquired from 500 *m/z* – 4000 *m/z* in reflectron mode. The acceleration voltage was set at 20 kV and the pulsed-ion extraction was set at 200 ns. One thousand laser shots were acquired for each spectrum. External mass calibration was achieved using a standard peptide mixture of Angiotensin I and II, Substance P, Renin Substrate, and ACTH (Bruker Daltonics Inc.). The externally calibrated mass accuracy of the instrument was approximately 100 parts-per-million (ppm) at *m/z* 1500.

### MALDI-TOF/TOF MS/MS analysis for identification of detected peptides from single brain on-target extraction sample preparation

Fragmentation spectra were acquired in LIFT mode on an autoflex III and an ultraflex III MALDI-TOF/TOF mass spectrometer (Bruker Daltonics Inc., Billerica, MA). MS spectra were acquired in positive ion and reflectron modes. For MS/MS analysis, the source acceleration voltage was set to 8.0 kV and the reflectron voltage was set to 29.5 kV. Mass spectra were acquired with approximately 3000 laser shots summed in 200 to 400 shot increments. External mass calibration was achieved using a standard peptide mixture of Angiotensin I and II, Substance P, Renin Substrate, and ACTH (Bruker Daltonics Inc.). The external calibration mass accuracy of the instrument was approximately 20 ppm in MS mode and <400 ppm in LIFT (MS/MS) mode. MS/MS spectra were not internally calibrated. All spectra were processed with FlexAnalysis software (Bruker Daltonics Inc.).

### Individual fly brain MALDI-TOF MS profiling data processing and statistical analysis

For preprocessing and statistical comparison of the relative abundance of PDF from MALDI-TOF profile spectra, all spectra obtained were first realigned by internal calibration using a mass list of neuropeptides we identified with MALDI-TOF/TOF MS/MS that were typically observed in our profile spectra. Internally calibrated spectra were exported to a plain text, two-column (*m/z* sampling points and corresponding intensities) ASCII format so they could be loaded into MATLAB 2013a (Mathworks, Natick, MA, USA). The msresample function in the MATLAB Bioinformatics Toolbox was used to resample internally calibrated spectra to a uniformly-spaced common set of *m/z* axis values.

Spectra from the two repeat experiments were analyzed separately. Spectra were denoised [40] in MATLAB using the undecimated discrete wavelet transform (UDWT) found in the Rice Wavelet Toolbox (http://www.dsp.ece.rice.edu/software/rwt.shtml) with a Daubechies’ scaling filter of length 8, soft thresholding applied, and the thresholding of low pass components enabled. Spectra were then baseline subtracted using the msbackadj function in the MATLAB Bioinformatics Toolbox with the default settings for this function. A total average spectrum of the denoised/baseline-subtracted spectra across all analyzed samples (including across conditions) within an experimental repeat was calculated. The total average spectrum was then normalized to its greatest value (i.e. the base peak). The mspeaks function from the MATLAB Bioinformatics Toolbox was then used to identify peaks from the total average spectrum, with the HeightFilter option of the mspeaks function set to “0.01”, so that only local maxima of the total average spectrum with intensity greater than 1% of the total average spectrum’s base peak are identified as peaks. When applying the mspeaks function, the left and right *m/*z locations of the full width at half height (FWHH) limits for each identified peak in the total average spectrum was specified to be returned. These values were used to establish peak bins.

Individual denoised and baseline-subtracted spectra were additionally normalized by dividing each point in the spectrum by the sum of all intensities in the spectrum after denoising and baseline subtraction (i.e. a total ion current normalization, although we avoid the term here to specify total area under the spectrum is taken after denoising and baseline-subtraction). Peak bins from the total average spectrum were used to query across each spectrum being analyzed, with the maximum value in each peak bin returned for each spectrum. This reduced the set of all spectra being processed into a single n × m matrix, the entries of one dimension representing the n peaks found by mspeaks and the entries of the second dimension corresponding to the m spectra being analyzed.

Intensity values taken from certain peak bins were further added together by combining peak bins that corresponded to isotope distributions of the same singly charged ion. Criteria for combining peak bins corresponding to the same isotope distribution required that at least three peaks be detected with centroided *m*/*z*’s differing by 1 ± 0.03, with the peak corresponding to the monoisotopic peak in a distribution being the most abundant ion mass in the total average spectrum for distributions less than 1700 *m/z* and the second peak in a distribution always having to be more abundant than the third and fourth peak in the total average spectrum. Series of peaks meeting this criteria were assigned as “isotopically resolved”.

Non-parametric statistical analysis (Kruskal-Wallis one-way ANOVA) was performed in MATLAB, comparing the sum of the maximum intensities of isotopically resolved distributions in each spectrum across the three fly strains being analyzed. The *p*-value obtained from Kruskal-Wallis one-way ANOVA for each isotopically resolved distribution was adjusted using a simple Bonferroni correction based on the total number of isotope distributions being statistically compared so that a significance level of α = 0.01 could be used despite multiple comparisons. Tukey’s least significant difference procedure (α = 0.05) was used as a post-hoc analysis to evaluate differences between conditions of isotopically resolved peaks found to be significantly different in the ANOVA analysis.

### MALDI-TOF MS/MS data processing for neuropeptide identification

MALDI-TOF/TOF MS/MS fragmentation spectra were exported to the FlexAnalysis software for batch preprocessing consisting of top-hat baseline subtraction; smoothing with four, width 0.15 m/z, Savitzky-Golay cycles; and peak picking using the SNAP algorithm with averagine molecular composition. All MS/MS spectra were combined and exported from FlexAnalysis as a single Mascot (Matrix Science Inc., London, England) generic file (*.mgf). The mgf file was submitted to an in-house Mascot server (version 2.2.07) for putative peptide identification using a 0.15 Da cutoff for precursor and a 0.5 Da cutoff for MS/MS peaks. Spectra were searched against the SwissProt 56.0 database with taxonomy specified as *Drosophila* (including 5357 protein sequences). Parameters for the Mascot search included enzyme specified as “none”, and variable modifications (C-terminal amidation, N-terminal pyroglutamic acid modification, methionine oxidation, and tyrosine sulfation) were considered. FlexAnalysis preprocessed MS/MS data were also moved to the BioTools software program (Bruker Daltonics Inc.) for manual confirmation of Mascot’s peptide assignments. A putative ID was considered confirmed when at least 3 consecutive b- or y-ions were observed, and in addition, the majority of MS/MS peaks were assigned. Although Mascot is tuned for protein-, not peptide-level identification (its significance scores at the peptide level are conservative), further confirmation came from the Mascot scoring algorithm in the form of a peptide score, peptide rank, and expectation value. For example, 13 of the 14 manually confirmed peptide identifications were also the highest ranking peptide from the Mascot search, and six of the 14 manually assigned peptides had Mascot Scores in the statistically significant range for protein identification.

### MALDI-FTICR-MS

MALDI-Fourier transform ion cyclotron resonance (FTICR)-MS was performed on an Apex Qe ultra 7 Tesla MALDI-FT-ICR mass spectrometer (Bruker Daltonics Inc., Billerica, MA). Mass spectra were collected in positive ion mode. The external mass accuracy of the instrument was approximately 20 ppm. After internal calibration, mass accuracy ranged from 0–2 ppm with a mean value of 0.5 ppm. Mass calibration was achieved using a standard peptide mixture of Angiotensin I and II, Substance P, Renin Substrate, and ACTH (Bruker Daltonics Inc.). Spectra were processed with DataAnalysis software (Bruker Daltonics Inc.).

## Abbreviations

MS: Mass spectrometry; MS/MS: Tandem mass spectrometry; MALDI: Matrix-assisted laser desorption/ionization; TOF: Time-of-flight; ppm: Part-per-million; FTICR: Fourier transform ion cyclotron resonance; PDF: Neuropeptide pigment-dispersing factor; NPLP1: Neuropeptide-like precursor 1; α-CHCA: α-cyano-4-hydroxycinnamic acid.

## Competing interests

The authors declare that they have no competing interests.

## Authors’ contributions

JNA and MR designed the experiment; KJB and YAH performed the experiments, with further contributions by JQ and AS; GG contributed the UAS-*Drm-pdf* line. PJK and MLE assisted with instrumentation. JNA, KJB, and JPS analyzed the data, prepared the figures, and wrote the manuscript. All authors have read and given approval to the final version of the manuscript.

## Supplementary Material

Additional file 1: Figure S1 On-target extraction provides spectra with greater signal-to-noise and more peaks from the region surrounding the tissue, as opposed to acquiring spectra directly from the tissue. **A)** Acquiring spectra from directly over the deposited *D. melanogaster* brain (shown at the center of the crosshair encircled in red) did not provide quality spectra reliably. Rather, the region outside the red circle, which made up the visible matrix spot encircled approximately in orange, was where the best signal was obtained. **B)** Shows the same regions encircled with the crosshairs positioned over an area representative of a region that provides high and varied ion signal in the peptide mass range. Click here for file

Additional file 2: Table S1 This file contains supporting material, including the following tables. A complete list of isotopically resolved ion masses detected in either experiment (with neuropeptide assignments when possible), intensity means with standard deviations calculated within each group for each of these ion masses, and the adjusted *p*-values from Kruskal-Wallis ANOVA. **Table S2.** Correlation between experiments of fourteen neuropeptides confirmed by MS/MS fragmentation. **Table S3.** Correlation between experiments of all isotopically resolved ion signals observed in both experiments. **Table S4.** Correlation between [M + H]^+^ and [M + K]^+^ of neuropeptide PDF within experimental conditions for both repeat experiments. Format: XLSX (Excel Spreadsheet); Size: 45 KB. Click here for file

## References

[B1] Baggerman G Boonen K Verleyen P De Loof A Schoofs L Peptidomic analysis of the larval Drosophila melanogaster central nervous system by two-dimensional capillary liquid chromatography quadrupole time-of-flight mass spectrometry J Mass Spectrom 2005 6 2 250 260 10.1002/jms.744 15706625

[B2] Altstein M Nassel DR Neuropeptide signaling in insects Adv Exp Med Biol 2010 6 155 165 10.1007/978-1-4419-6902-6_8 21189678

[B3] Clynen E Reumer A Baggerman G Mertens I Schoofs L Neuropeptide biology in Drosophila Adv Exp Med Biol 2010 6 192 210 10.1007/978-1-4419-6902-6_10 21189680

[B4] Strand FL New vistas for melanocortins. Finally, an explanation for their pleiotropic functions Ann N Y Acad Sci 1999 6 1 16 10.1111/j.1749-6632.1999.tb07874.x 10676431

[B5] Merighi A Salio C Ferrini F Lossi L Neuromodulatory function of neuropeptides in the normal CNS J Chem Neuroanat 2011 6 4 276 287 10.1016/j.jchemneu.2011.02.001 21385606

[B6] Neupert S Johard HA Nassel DR Predel R Single-cell peptidomics of drosophila melanogaster neurons identified by Gal4-driven fluorescence Anal Chem 2007 6 10 3690 3694 10.1021/ac062411p 17439240

[B7] Baggerman G Cerstiaens A De Loof A Schoofs L Peptidomics of the larval Drosophila melanogaster central nervous system The Journal of biological chemistry 2002 6 43 40368 40374 10.1074/jbc.M206257200 12171930

[B8] Schoofs L Baggerman G Peptidomics in Drosophila melanogaster Brief Funct Genomic Proteomic 2003 6 2 114 120 10.1093/bfgp/2.2.114 15239932

[B9] Predel R Wegener C Russell WK Tichy SE Russell DH Nachman RJ Peptidomics of CNS-associated neurohemal systems of adult Drosophila melanogaster: a mass spectrometric survey of peptides from individual flies J Comp Neurol 2004 6 3 379 392 10.1002/cne.20145 15174081

[B10] Hewes RS Taghert PH Neuropeptides and neuropeptide receptors in the Drosophila melanogaster genome Genome Res 2001 6 6 1126 1142 10.1101/gr.169901 11381038 PMC311076

[B11] Vanden Broeck J Neuropeptides and their precursors in the fruitfly, Drosophila melanogaster Peptides 2001 6 2 241 254 10.1016/S0196-9781(00)00376-4 11179818

[B12] Pyza E Meinertzhagen IA The regulation of circadian rhythms in the fly’s visual system: involvement of FMRFamide-like neuropeptides and their relationship to pigment dispersing factor in Musca domestica and Drosophila melanogaster Neuropeptides 2003 6 5 277 289 10.1016/j.npep.2003.06.001 14607105

[B13] Wang C Zhang J Tobe SS Bendena WG Defining the contribution of select neuropeptides and their receptors in regulating sesquiterpenoid biosynthesis by Drosophila melanogaster ring gland/corpus allatum through RNAi analysis Gen Comp Endocrinol 2012 6 3 347 353 10.1016/j.ygcen.2011.12.039 22245290

[B14] Renn SC Park JH Rosbash M Hall JC Taghert PH A pdf neuropeptide gene mutation and ablation of PDF neurons each cause severe abnormalities of behavioral circadian rhythms in Drosophila Cell 1999 6 7 791 802 10.1016/S0092-8674(00)81676-1 10619432

[B15] Hummon AB Richmond TA Verleyen P Baggerman G Huybrechts J Ewing MA Vierstraete E Rodriguez-Zas SL Schoofs L Robinson GE From the genome to the proteome: uncovering peptides in the Apis brain Science 2006 6 5799 647 649 10.1126/science.1124128 17068263

[B16] Li B Predel R Neupert S Hauser F Tanaka Y Cazzamali G Williamson M Arakane Y Verleyen P Schoofs L Genomics, transcriptomics, and peptidomics of neuropeptides and protein hormones in the red flour beetle Tribolium castaneum Genome Res 2008 6 1 113 122 18025266 10.1101/gr.6714008 PMC2134770

[B17] De Loof A Ecdysteroids, juvenile hormone and insect neuropeptides: Recent successes and remaining major challenges Gen Comp Endocrinol 2008 6 1 3 13 10.1016/j.ygcen.2007.07.001 17716674

[B18] Carlsson MA Diesner M Schachtner J Nassel DR Multiple neuropeptides in the Drosophila antennal lobe suggest complex modulatory circuits J Comp Neurol 2010 6 16 3359 3380 10.1002/cne.22405 20575072

[B19] Yew JY Wang Y Barteneva N Dikler S Kutz-Naber KK Li L Kravitz EA Analysis of Neuropeptide expression and localization in adult drosophila melanogaster central nervous system by affinity cell-capture mass spectrometry J Proteome Res 2009 6 3 1271 1284 10.1021/pr800601x 19199706 PMC2693453

[B20] Overend G Cabrero P Guo AX Sebastian S Cundall M Armstrong H Mertens I Schoofs L Dow JA Davies SA The receptor guanylate cyclase Gyc76C and a peptide ligand, NPLP1-VQQ, modulate the innate immune IMD pathway in response to salt stress Peptides 2012 6 1 209 218 10.1016/j.peptides.2011.08.019 21893139

[B21] Brockmann A Annangudi SP Richmond TA Ament SA Xie F Southey BR Rodriguez-Zas SR Robinson GE Sweedler JV Quantitative peptidomics reveal brain peptide signatures of behavior Proc Natl Acad Sci USA 2009 6 7 2383 2388 10.1073/pnas.0813021106 19179284 PMC2632711

[B22] Chen R Hui L Cape SS Wang J Li L Comparative neuropeptidomic analysis of food intake via a multi-faceted mass spectrometric approach ACS Chem Neurosci 2010 6 3 204 214 10.1021/cn900028s 20368756 PMC2847303

[B23] Frese CK Boender AJ Mohammed S Heck AJR Adan RAH Altelaar AFM Profiling of diet-induced Neuropeptide changes in Rat brain by quantitative mass spectrometry Anal Chem 2013 6 9 4594 4604 10.1021/ac400232y 23581470

[B24] Christie AE Stemmler EA Peguero B Messinger DI Provencher HL Scheerlinck P Hsu YW Guiney ME de la Iglesia HO Dickinson PS Identification, physiological actions, and distribution of VYRKPPFNGSIFamide (Val1)-SIFamide) in the stomatogastric nervous system of the American lobster Homarus americanus J Comp Neurol 2006 6 3 406 421 10.1002/cne.20932 16566002

[B25] Garden RW Sweedler JV Heterogeneity within MALDI samples as revealed by mass spectrometric imaging Anal Chem 2000 6 1 30 36 10.1021/ac9908997 10655631

[B26] Brand AH Perrimon N Targeted gene expression as a means of altering cell fates and generating dominant phenotypes Development 1993 6 2 401 415 8223268 10.1242/dev.118.2.401

[B27] Hou X Xie F Sweedler JV Relative quantitation of neuropeptides over a thousand-fold concentration range J Am Soc Mass Spectrom 2012 6 12 2083 2093 10.1007/s13361-012-0481-0 22993045 PMC3515743

[B28] Rubakhin SS Sweedler JV Quantitative measurements of cell-cell signaling peptides with single-cell MALDI MS Anal Chem 2008 6 18 7128 7136 10.1021/ac8010389 18707135 PMC2646760

[B29] Morris JS Coombes KR Koomen J Baggerly KA Kobayashi R Feature extraction and quantification for mass spectrometry in biomedical applications using the mean spectrum Bioinformatics 2005 6 9 1764 1775 10.1093/bioinformatics/bti254 15673564

[B30] Wegener C Reinl T Jansch L Predel R Direct mass spectrometric peptide profiling and fragmentation of larval peptide hormone release sites in Drosophila melanogaster reveals tagma-specific peptide expression and differential processing J Neurochem 2006 6 5 1362 1374 10.1111/j.1471-4159.2005.03634.x 16441518

[B31] Czyzyk TA Ning Y Hsu MS Peng B Mains RE Eipper BA Pintar JE Deletion of peptide amidation enzymatic activity leads to edema and embryonic lethality in the mouse Dev Biol 2005 6 2 301 313 10.1016/j.ydbio.2005.09.001 16225857

[B32] Kolhekar AS Roberts MS Jiang N Johnson RC Mains RE Eipper BA Taghert PH Neuropeptide amidation in Drosophila: separate genes encode the two enzymes catalyzing amidation J Neurosci 1997 6 4 1363 1376 9006979 10.1523/JNEUROSCI.17-04-01363.1997 PMC6793724

[B33] Verleyen P Baggerman G Wiehart U Schoeters E Van Lommel A De Loof A Schoofs L Expression of a novel neuropeptide, NVGTLARDFQLPIPNamide, in the larval and adult brain of Drosophila melanogaster J Neurochem 2004 6 2 311 319 14690519 10.1046/j.1471-4159.2003.02161.x

[B34] Romanova EV Lee JE Kelleher NL Sweedler JV Gulley JM Comparative peptidomics analysis of neural adaptations in rats repeatedly exposed to amphetamine J Neurochem 2012 6 2 276 287 10.1111/j.1471-4159.2012.07912.x 22860605 PMC3463764

[B35] Hanrieder J Wicher G Bergquist J Andersson M Fex-Svenningsen A MALDI mass spectrometry based molecular phenotyping of CNS glial cells for prediction in mammalian brain tissue Anal Bioanal Chem 2011 6 1 135 147 10.1007/s00216-011-5043-y 21553124

[B36] Hettick JM Kashon ML Simpson JP Siegel PD Mazurek GH Weissman DN Proteomic profiling of intact mycobacteria by matrix-assisted laser desorption/ionization time-of-flight mass spectrometry Anal Chem 2004 6 19 5769 5776 10.1021/ac049410m 15456297

[B37] Park JH Helfrich-Forster C Lee G Liu L Rosbash M Hall JC Differential regulation of circadian pacemaker output by separate clock genes in Drosophila Proc Natl Acad Sci U S A 2000 6 7 3608 3613 10.1073/pnas.97.7.3608 10725392 PMC16287

[B38] Park JH Hall JC Isolation and chronobiological analysis of a neuropeptide pigment-dispersing factor gene in Drosophila melanogaster J Biol Rhythms 1998 6 3 219 228 10.1177/074873098129000066 9615286

[B39] Rio DC Rubin GM Transformation of cultured Drosophila melanogaster cells with a dominant selectable marker Mol Cell Biol 1985 6 8 1833 1838 3018529 10.1128/mcb.5.8.1833-1838.1985 PMC366898

[B40] Coombes KR Tsavachidis S Morris JS Baggerly KA Hung MC Kuerer HM Improved peak detection and quantification of mass spectrometry data acquired from surface-enhanced laser desorption and ionization by denoising spectra with the undecimated discrete wavelet transform Proteomics 2005 6 16 4107 4117 10.1002/pmic.200401261 16254928

