# Supplementary figures and images for: A rapid MALDI-TOF mass spectrometry workflow for Drosophila melanogaster differential neuropeptidomics
Source: Mol Brain. 2013 Dec 27;6:60. doi: 10.1186/1756-6606-6-60 (PMC4022047; doi:10.1186/1756-6606-6-60)

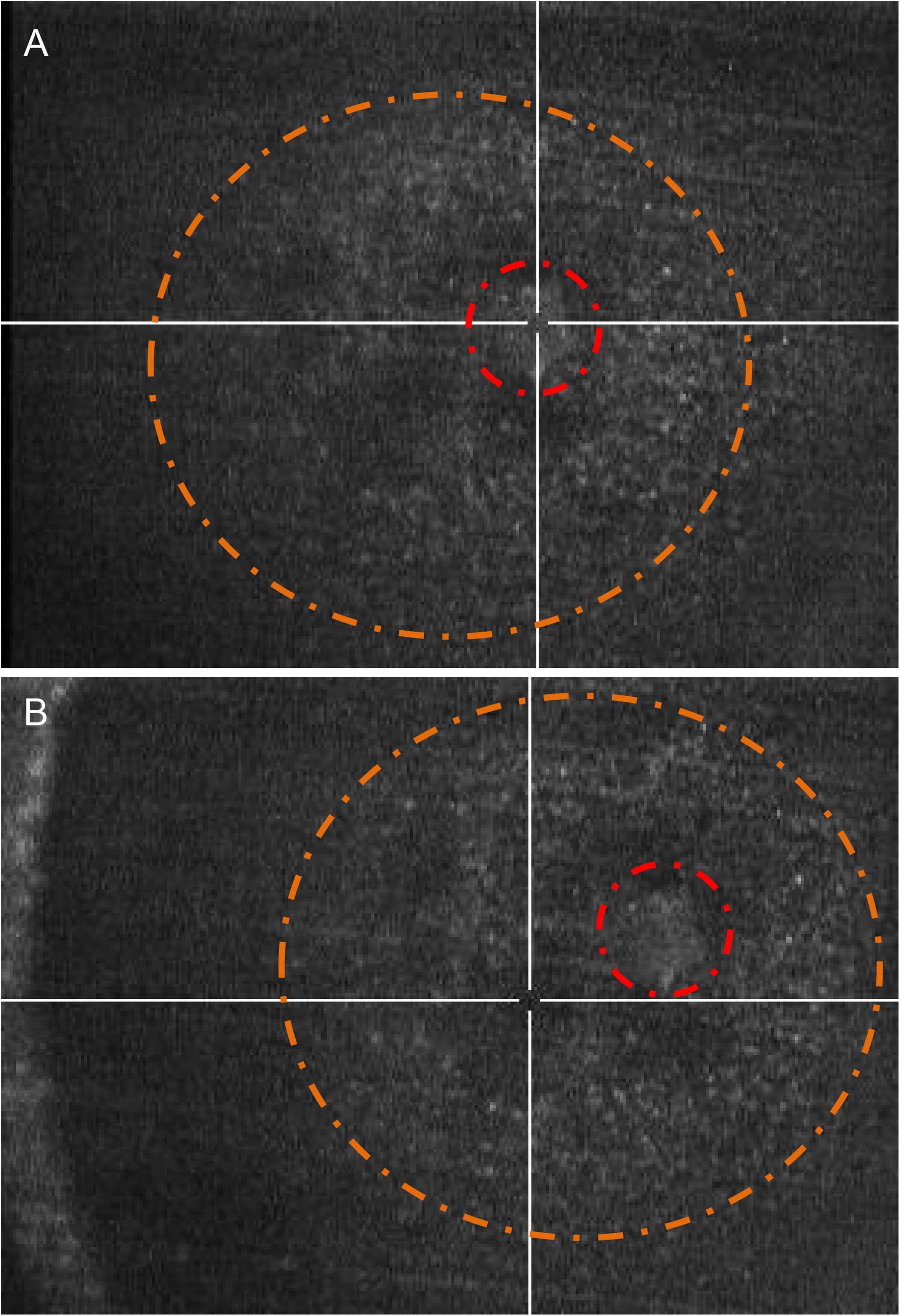

Supplement: Additional file 1: Figure S1 — On-target extraction provides spectra with greater signal-to-noise and more peaks from the region surrounding the tissue, as opposed to acquiring spectra directly from the tissue. A) Acquiring spectra from directly over the deposited D. melanogaster brain (shown at the center of the crosshair encircled in red) did not provide quality spectra reliably. Rather, the region outside the red circle, which made up the visible matrix spot encircled approximately in orange, was where the best signal was obtained. B) Shows the same regions encircled with the crosshairs positioned over an area representative of a region that provides high and varied ion signal in the peptide mass range. [file 1756-6606-6-60-S1.tiff]
